# Not all observed actions are perceived equally

**DOI:** 10.1038/s41598-017-17369-z

**Published:** 2017-12-06

**Authors:** Artem Platonov, Guy A. Orban

**Affiliations:** Department of Medicine and Surgery, University of Parma, Parma, Italy

## Abstract

Action observation is the visual process analyzing the actions of others to determine their goals and how the actor’s body (part) movements permit attaining those goals. Our recent psychophysical study demonstrated that 1) observed action (OA) perception differs from shape perception in viewpoint and duration dependence, and 2) accuracy and reaction times of OA discrimination are fitted by the proportional-rate diffusion model whereby a sensory stage provides noisy evidence that is accumulated up to a criterion or bound by a decision stage. That study was devoted to observation of manipulative actions, following a general trend of the field. Recent functional imaging studies of action observation, however, have established various OA classes as separate entities with processing routes involving distinct posterior parietal cortex (PPC) regions. Here, we show that the diffusion model applies to multiple OA classes. Even more importantly, the observers’ ability to discriminate exemplars of a given class differs considerably between OA classes and these performance differences correspond to differences in model parameters. In particular, OA classes differ in the bound parameter which we propose may reflect an urgency signal originating in the PPC regions corresponding to the sensory stages of different OA classes.

## Introduction

**Figure 1:**
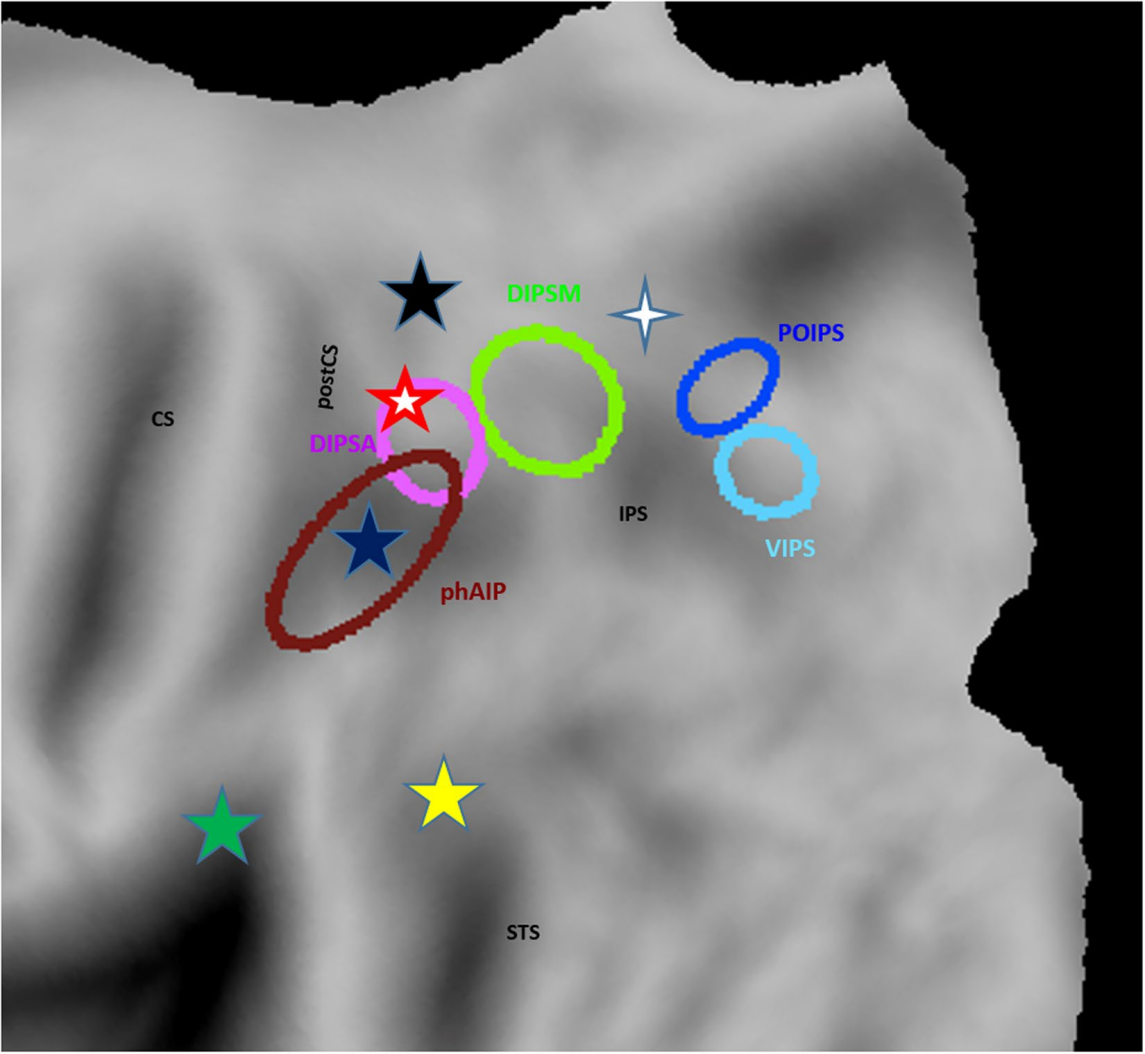
PPC regions specifically involved in action observation. Partial view of left flattened hemisphere with position of regions involved in observing manipulation (blue star^5,7,8^), skin-displacement (green star^7^), vocal communication (yellow star^8^), climbing (black star^5^), interpersonal actions (red/white star^7^) and reaching (blue/white star^43^). The ellipses indicate the confidence limits defining putative human anterior intraparietal (phAIP) area, dorsal intraparietal sulcus anterior (DIPSA), dorsal intraparietal sulcus medial (DIPSM), parieto-occipital intraparietal sulcus (POIPS) and ventral intraparietal sulcus (VIPS) regions^44^. Not only do these regions differ in location, they also differ in extent. For the three action classes considered here, observing climbing specifically activated 661 voxels, observing manipulation 406 voxels and observing skin-displacing actions a mere 70 voxels, using the number of voxels reaching p < 0.05 in the interaction to estimate the extent.

Action observation refers to the visual process of assessing the goals of actions performed by conspecifics, and how the effector movements allow achieving those goals^1^. Our recent psychophysical investigation of discrimination between two manipulative hand actions demonstrated that observed action (OA) perception differs from shape perception in viewpoint and duration dependence^1^. In addition, accuracy and reaction times in that study were well described by the proportional-rate diffusion model whereby a sensory stage provides noisy evidence that is accumulated up to a criterion or bound by a decision stage. Moreover, recent developments regarding action observation have established various OA classes as separate entities with processing routes involving distinct posterior parietal cortex (PPC) regions (Fig. 1). Here, we show that the diffusion model applies to multiple OA classes. Even more importantly, the differences in observers’ ability to discriminate exemplars of the various action classes correspond to differences in model parameters between classes. In particular, OA classes differ in the bound parameter which we propose may reflect an urgency signal originating in the PPC regions corresponding to the sensory stages of different OA classes.

So far, action observation studies have been heavily biased towards grasping and to a lesser degree manipulative hand actions^2^. Although the exact taxonomy of human actions is far from firmly established, distinctions between observed action (OA) classes becomes increasingly clear^3^. The basis for defining observed action classes are the underlying sensorimotor transformations. For example, the sensory information required for planning hand manipulation are object properties such as size, shape and weight, which differ from the 3D layout of the environment needed for locomotion. Because these sensory cues are processed in different parts of PPC, which also houses a coarse map of effectors^4^, the various sensorimotor transformations are computed in different PPC regions. Based on a host of imaging studies^2^, we have hypothesized^3^ that the observed actions are processed in the same PPC regions as those where actions are planned, and hence, that OA classes engage different PPC regions. This prediction has been borne out by our recent imaging studies: observing locomotion specifically activates a rostro-dorsal SPL site^5^, manipulation phAIP^5–8^ and skin displacing actions a small region straddling SII and PFop (Fig. 1).

Differences in the neural organization at higher stages of the processing of OA classes raises two psychophysical questions that we address here. First, we need to know whether or not results of manipulative hand action discrimination experiments generalize to other action classes. Thus, as a first goal, this study shall test the applicability of the diffusion model to OA classes other than manipulative hand actions. Three parameters (bound, drift rate, and residual time) characterize the diffusion model, in which a decision process continuously accumulates noisy evidence over time towards one of two possible decision criteria (*bounds*). Reaching one of these bounds triggers the response corresponding to this bound. The rate at which stimulus information accumulates (*drift rate*) is determined by the relative amount of information that is absorbed per unit time and can be interpreted as a measure of perceptual sensitivity, in a between-person comparison, or as a measure of task difficulty, in a between-condition comparison^9^. Bounds control the amount of information needed by a decision-maker to commit to one of the alternatives and are typically described in terms of a speed versus accuracy trade-off^9,10^. The response time also includes time taken by non-decision-related components, defining the third parameter: *residual time*.

On the other hand, the existence of different organizations at the action observation network level also invite an investigation of psychophysical differences between OA classes both at performance and model parameter levels. So far, studies testing the applicability of diffusion models in the visual domain have typically used simple visual features such as brightness, color, numerosity (for review see^11^), or motion-direction discrimination (for review see^12^). While these studies have shown that parameters of the diffusion model can be affected by procedural manipulations (e.g. instructions altering the speed accuracy tradeoff) or participants’ age, stimulus manipulation produced but modest effects except for changes in stimulus strength which influenced drift rate and frequency of presentation modifying residual time. One could expect greater differences between model parameters for OA classes because of the ecological validity of the action videos compared to the mostly artificial stimuli used in previous visual discrimination studies. Thus, comparing psychophysical performance and model parameters for different OA classes was the second goal of the present study. For the OA classes to be investigated, we selected locomotion and skin-displacing actions, as they activate very different regions in PPC (Fig. 1) and involve very different typical effectors: lower limbs for locomotion and upper limb for skin-displacing actions. Results were compared to those obtained earlier^1^ for manipulation, allowing us to contrast three classes.

We tested the discrimination of skin-displacing actions (experiment 1, Supplementary video 1) and locomotion (experiment 2, Supplementary video 2) using a two-alternative forced-choice (2AFC) action discrimination task. In each experiment, 10 subjects viewed a single video clip portraying an action exemplar and, as in the previous study^1^, indicated their choice between two exemplars by pressing one of two buttons with the right hand as soon as they were ready. Since the comparison of the performance of the five subjects who participated in the previous action discrimination experiment^1^ and the five totally naïve subjects did not reveal any differences (see supplementary materials), we pooled data from these 2 groups for all further analysis. The proportional-rate diffusion model was fitted to the accuracies and response times acquired in the experiments^1,13^. As before^1^, we tested the validity of the assumption made in the modeling that the bounds (*A’*) are symmetrical relative to the starting point, meaning that subjects show no bias towards either of the two alternatives^10^. Therefore, in both experiments, we calculated the response bias (c) after Macmillan & Creelman^14^ and found it to be very small (c < 10^−15^) in all subjects (N = 20) and all signal strength conditions. Hence, as before^1^, to facilitate comparison of our results with those of Platonov and Orban^1^, we expressed performance as a single variable, accuracy, ranging from 50 to 100%, by combining responses to the two action exemplars. In what follows, we integrate the analysis of the 2 OA classes tested in the present study with results for the class of manipulative hand actions (rolling and rotation action exemplars) obtained with nine subjects participating in experiment 1 of Platonov & Orban^1^.

**Figure 2:**
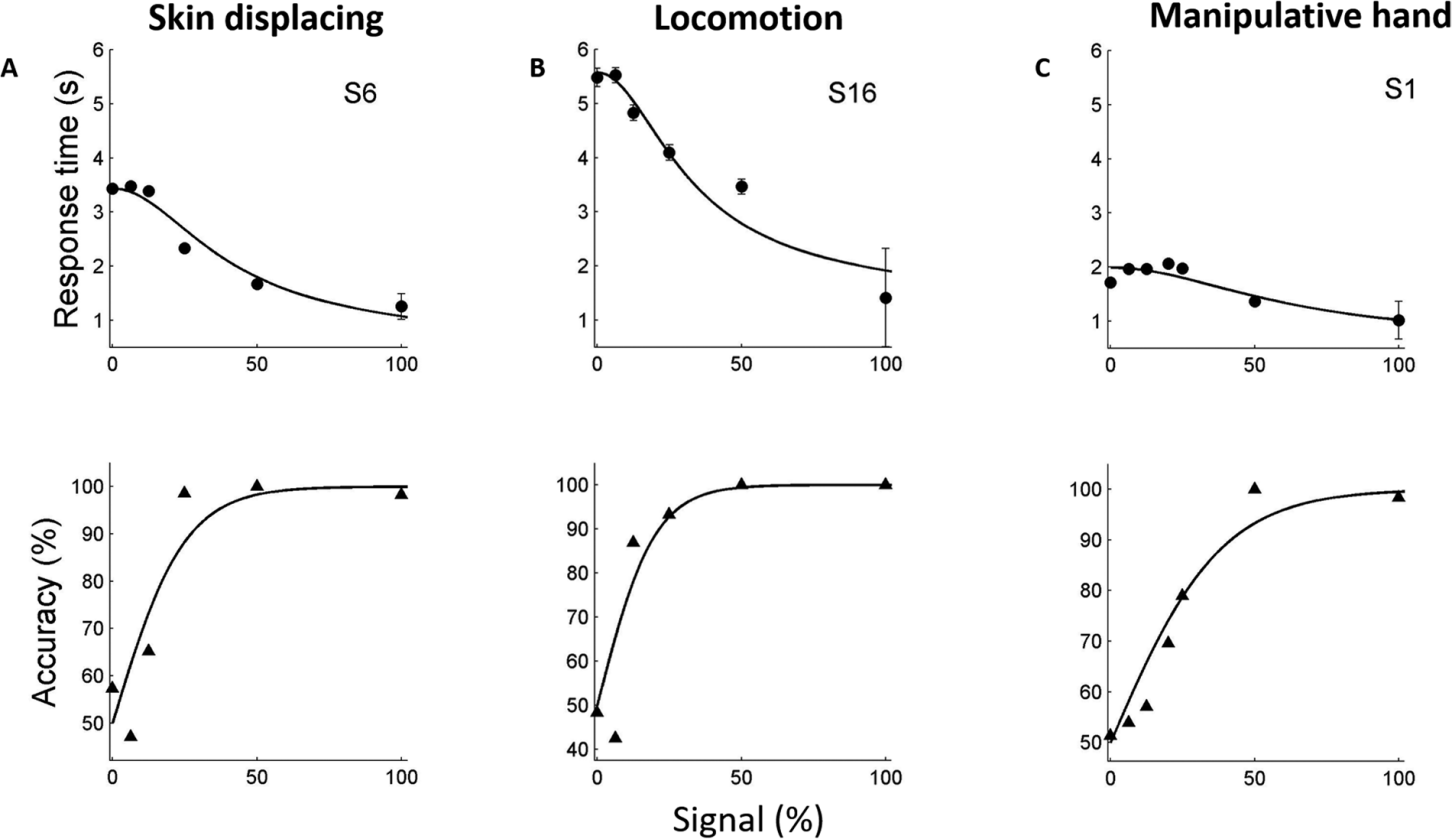
Single subject response time (circles, upper rows) and accuracy (triangles, lower rows) plotted as a function of signal strength for 2AFC discrimination of observed skin displacement (**A**), locomotion (**B**) and manipulative hand (**C**, borrowed from^1^) actions. The proportional-rate diffusion model provided a close fit (solid lines) to the data in all subjects. Error bars indicate ±1 SEM.

Figure 2 plots the results from single subjects discriminating observed skin displacing (Fig. 2A), locomotion (Fig. 2B) and manipulative hand actions (Fig. 2C, taken from^1^) for accuracy (triangles) and response times (circles). Similar results are shown for all other subjects in Supplementary Figures 1 and 2, respectively. One of the most striking differences in data derived from different OA classes are the response times at 0% signal. These average response time values were about twice as long for locomotion (average response time 4.50 s ± 0.06) compared to skin displacing (average response time 2.90 s ± 0.07) and manipulative hand (average response time 2.13 s ± 0.05) actions. Yet the lowest values of response time at 100% signal were rather similar, averaging 1.37 s ± 0.01 for locomotion, compared to 1.14 s ± 0.03 for the skin displacement and 1.18 ± 0.03 for manipulative hand actions.

The diffusion model predicts that this discrepancy in response times is a consequence of an elevated bound for the locomotion action class compared to other 2 action classes^10^. Hence, as the next step, we fitted the proportional-rate diffusion model with 3 free-parameters^10^ to the novel data (Fig. 2, solid lines; Supplementary Figures 1 and 2, solid lines; Supplementary Table 2). As in the previous study^1^, the robust correlations (Supplementary Figures 3 and 4) between predicted and measured accuracy, clearly different from zero (r = 0.88; t-test, p < 0.01 and r = 0.94; t-test, p < 0.01 for skin displacing and locomotion actions, respectively) demonstrated the close fit of the model to the data. The same held true for response time (r = 0.94; t-test, p < 0.01 and r = 0.94; t-test, p < 0.01 for skin displacing and locomotion actions, respectively). From the fitted model parameters, we calculated the halfway response time and 75% accuracy thresholds (Supplementary Table 2). These values were closely coupled with their ratios equaling about 3.5 in all subjects (Supplementary Table 2), as is characteristic of the proportional-rate diffusion model.

**Figure 3:**
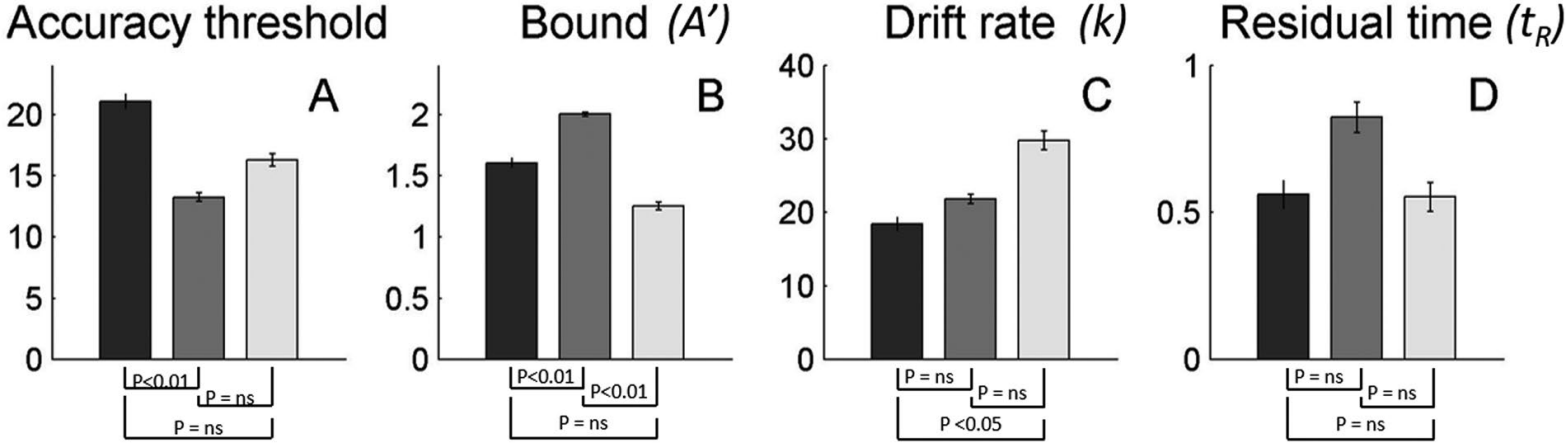
Accuracy threshold (**A**), bound (**B**), drift rate (**C**) and residual time (**D**) parameters and in skin displacing (black), locomotion (gray) and manipulative hand (light gray) action discrimination, averaged across the subjects. Accuracy thresholds in locomotion were significantly smaller than in skin-displacing actions (t-test, t (18) = 4.02, p < 0.01), while differences in accuracy thresholds between locomotion and skin-displacing actions (t-test, t (17) = 2.11, p > 0.05), on the one hand, and between skin-displacing and manipulative hand actions (t-test, t (17) = 2.19, p > 0.05), on the other, did not reach the level of significance. Locomotion actions were significantly different from skin-displacing actions in terms of bound parameter (t-test, t (18) = 3.67, p < 0.01), while the difference between skin displacing and manipulative hand actions (from^1^) did not reach the level of significance for bound (t-test, t (17) = 2.62, p > 0.05). Although difference in thresholds between locomotion and manipulative hand actions did not reach the level of significance, their bounds were significantly different (t-test, t (17) = 8.05, p < 0.01). Drift rate parameter was only different between skin displacing actions and manipulative hand actions (t-test, t (17) = 2.71, p < 0.05), whereas we found no difference between skin displacing and locomotion actions (t-test, t (18) = 1.11, p > 0.05), on the one hand, nor locomotion and manipulative hand actions (t-test, t (17) = 2.12, p > 0.05), on the other. There were also no differences in residual time parameters between 3 action classes (t-test, t (18) = 1.33, p > 0.05; t-test, t (18) = 1.35, p > 0.05; t-test, t (17) = 0.04, p > 0.05, for skin displacing versus locomotion actions, manipulative hand versus locomotion actions and manipulative hand versus skin displacing actions, respectively). Error bars indicate ±1 SEM.

The accuracy thresholds thus derived documented even clearer differences between OA classes (Fig. 3A). Indeed a one-way ANOVA indicated that accuracy threshold values were significantly different amongst the 3 OA classes (one-way ANOVA: F_2,52_ = 13.03, p < 0.01) with locomotion actions discrimination having lowest threshold (Fig. 3A, gray; 13.2 ± 0.34) values followed by manipulative hand (Fig. 3A, light gray; 16.3 ± 0.51) and skin displacing (Fig. 3A, black; 21.1 ± 0.59) actions. As expected from the response time data, the difference in accuracy between locomotion and skin displacing actions was significant (t-test, t (18) = 4.02, p < 0.01).

Supplementary Figure 5 shows that dynamic changes in the videos of the locomotion class were much greater than those of the skin displacing actions. The retinal sizes of these changes were estimated by the number of local motion vectors averaged over all video durations (Supplementary Figure 5A–C) of a class (see methods). Indeed, while the average number of local motion vectors was hardly different between exemplars within each action class (<1%), that number of vectors in the locomotion class (Supplementary Figure 5B) exceeded that of skin displacing (Supplementary Figure 5A) and manipulative hand actions (Supplementary Figure 5C) by factors about 1.5 and 3, respectively. Furthermore local motion vectors were stronger (reached larger speeds) in locomotion than in skin displacing or manipulative actions (Supplementary Figure 5D). This suggests that performance differences could be accounted for by differences in stimulus strength as captured by the local motion vectors. However, this account no longer holds when skin displacing actions are compared to manipulative hand actions. While the accuracy thresholds for skin-displacing actions came close to being-significantly greater (t-test, t (17) = 2.11, p > 0.05), the local motion vectors in skin displacing actions were about twice as numerous as those of manipulative hand actions and their strength was 3 times larger (Supplementary Figure 5). This is clearly opposite the predicted result if the stimulus differences accounted for difference in performance across action classes. Moreover, note that drift rate, a commonly accepted indicator of difficulty, was significantly greater for manipulative hand actions than for skin displacing actions (t-test, t (17) = 2.71, p < 0.05). If stimulus strength, captured by local motion vectors, facilitated discrimination between 2 actions, then this relationship would be expected to be exactly opposite, given the lower accuracy thresholds in manipulative hand actions.

Figure 3 displays free-parameter values derived from the model, averaged across the subjects: bound (Fig. 3B), drift rate (Fig. 3C) and residual time (Fig. 3D) estimated for observing skin displacing (black), locomotion (gray) and manipulative hand (light gray) actions. Two out of the 3 parameters, *A’* (one-way ANOVA: F_3,33_ = 20.5, p < 0.01) and *k* (one-way ANOVA: F_3,33_ = 6.30, p < 0.01), were significantly different amongst the 3 action classes, while no significant differences were found for the parameter *t*_*R*_ (one-way ANOVA: F_3,33_ = 1.43, p > 0.05) across action classes. Difference in parameters *A’* and *k* were to be expected since accuracy varied between action classes and the model posits that accuracy threshold is proportional to both *A’* and *k* parameters, approximately as 0.55/*kA’* ^10^. Comparing action classes against one another reveals that the average *A’* parameter for locomotion actions was significantly higher than for either skin displacing (t-test, t (18) = 4.00, p < 0.01) or manipulative hand (t-test, t (17) = 8.05, p < 0.01) actions. Differences in *A’* between skin displacing and manipulative hand actions was not significant (t-test, t (17) = 2.62, p > 0.05). Given that neither the *k* nor the *t*_*R*_ parameters were significantly different between skin displacing and locomotion actions (t-test, t (18) = 1.11, p > 0.27 and t (18) = 1.33, p > 0.19, respectively), or between manipulative hand actions and locomotion actions (t-test, t (17) = 2.11, p > 0.05 and t (17) = 1.35, p > 0.19, respectively), we concluded that higher accuracy in discriminating observed locomotion was primarily a consequence of the elevated bound. To further support this conclusion, we fitted the data from the experiment 1 and 2 with the nested model in which *k* was the only free parameter. Both *A’* and *t*_*R*_ were assigned the mean values calculated earlier by Platonov & Orban (2016) for manipulative hand actions. The ability of this reduced model to fit the data from experiment 1did not significantly differ from the original, fuller model in most of the subjects (all subjects except S6) (Supplementary Table 3). That was to be expected given that we did not find any significant differences between skin displacing and manipulative hand actions in terms of *A’* and *t*_*R*_ (see above). For locomotion actions, however, tested in the experiment 2, the original, fuller model provided a significantly better fit than the reduced model in all subjects (Supplementary Table 2). Also note that the differences between action classes reported in this study could not be explained by the temporal structure of the stimuli (see supplementary materials).

**Figure 4:**
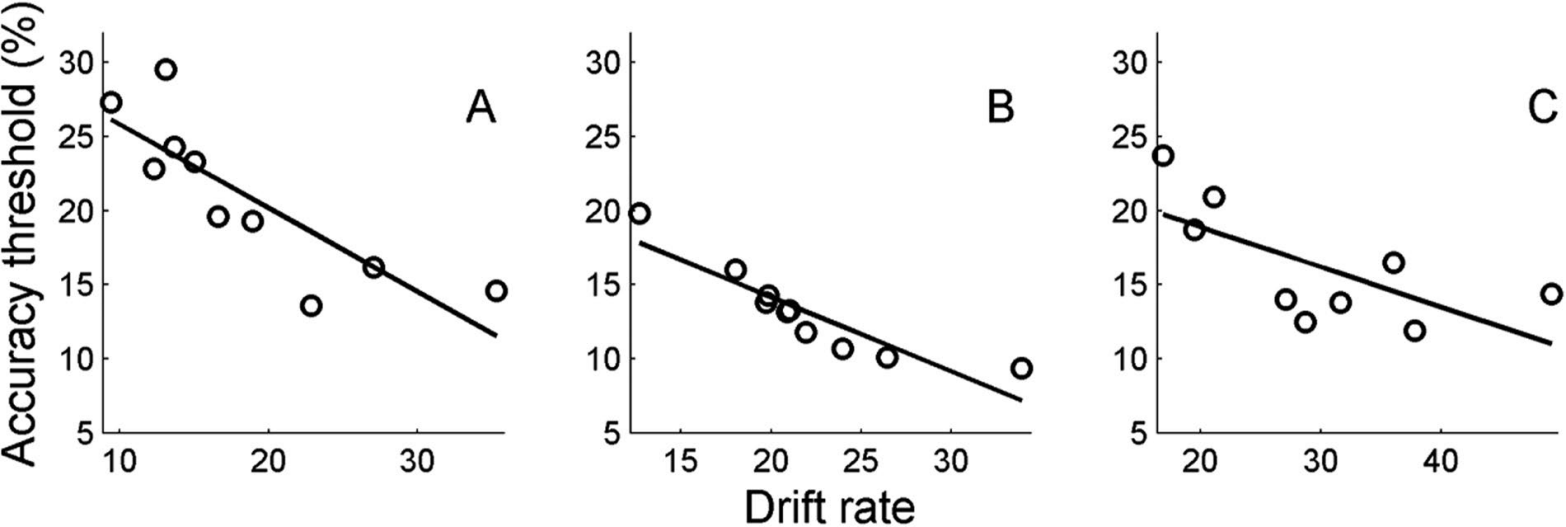
Scatterplot of drift rate versus accuracy thresholds calculated for skin displacing (**A**), locomotion (**B**) and manipulative hand (**C**) action discrimination. Bullet points identify the results from individual subjects. Lines are linear regression lines fitted to the data. There was a linear relationship between accuracy thresholds and k parameter in all 3 action classes (α = −0.56 ± 0.13, t(8) = 4.32, p < 0.01; α = −0.50 ± 0.08, t(8) = 6.14, p < 0.01 and α = −0.27 ± 0.11, t(7) = 2.46, p < 0.05, for the skin displacing, locomotion and manipulative hand actions, respectively).

Our results so far indicate that threshold differences in action discrimination for various OA classes are largely driven by different bound values characteristic for each OA class. On the other hand, inter-subject analysis revealed that, for all OA classes, between subjects threshold differences were largely unaffected by *A’* (correlation coefficients between *A’* and accuracy thresholds were r = 0.43, p > 0.21, r = 0.33, p > 0.35 and r = 0.11, p > 0.76 for the skin displacing, locomotion and manipulative hand actions, respectively). In addition, and unsurprisingly, residual time *t*_*R*_, was also unrelated to accuracy threshold values across the subjects (r = −0.09, p > 0.81, r = −0.67, p > 0.05 and r = 0.58, p > 0.10 for the skin displacing, locomotion and manipulative hand actions, respectively). The parameter defining variabilities in discrimination accuracy across subjects in all 3 OA classes was drift rate. The analysis indicated a significant negative modulation of the accuracy thresholds by *k* in skin displacing (r = −0.84, p < 0.01), locomotion (r = −0.91, p < 0.01) and manipulative hand (r = −0.68, p < 0.05) action discrimination. Figure 4 plots individual accuracy thresholds as a function of drift rate parameter for skin displacing (Fig. 4A), locomotion (Fig. 4B) and manipulative hand (Fig. 4C) actions. There was a linear relationship between accuracy thresholds and the *k* parameter with a significant negative slope in all 3 OA classes (α = −0.56 ± 0.13, t (8) = 4.32, p < 0.01; α = −0.50 ± 0.08, t (8) = 6.14, p < 0.01 and α = −0.27 ± 0.11, t (7) = 2.46, p < 0.05, for the skin-displacing, locomotion and manipulative hand actions, respectively).

Our data indicate that, in the 2AFC task, locomotion and skin displacing action classes elicit behavior similar to that earlier described for manipulative hand actions^1^. More specifically, a proportional-rate diffusion model provided a close fit to the experimental data for both novel action classes tested (Fig. 2, Supplementary Figures 1, 2) with high correlation between observed and predicted reaction times and accuracy (Supplementary Figures 3, 4). Moreover, the ratios of halfway response times and accuracy thresholds for the different action exemplar-pairs were very close to 3.5, the value typical of a diffusion process (Supplementary Table 2). Our results thus imply that the two-stage process assumed by the model, applies to the different OA classes. In this process the activity of neurons selective for specific action exemplars provides fluctuating sensory evidence, which accumulates at a second stage, at which a decision is made once a criterion bound is reached by the accumulated, noisy evidence. These findings extend to additional action classes the notion put forward by Platonov & Orban^1^ that observed actions are processed by neurons selective for the exemplars of these classes and deemed observed-action selective (OAS) neurons^15,16^. They further imply that OAS neurons selective for exemplars of different OA classes are housed in distinct PPC regions.

In addition, the present results indicate significant differences exist between the novel 2 action classes tested in this study, both of which also differ from the manipulative hand actions class reported earlier^1^. Most notably, thresholds for discriminating skin displacement exceeded those for manipulative hand actions and even more so those for locomotion. Importantly, results from our previous study showed that diffusion model parameters and calculated thresholds do not significantly differ between exemplar pairs within a given action class^1^. The ranking of the thresholds across OA classes matches, at least qualitatively, the sizes of the specific PPC regions dedicated to a specific action class, whereby larger areal sizes, presumably populated by more neurons, yield lower thresholds. Indeed, activation maps for observed action classes^5,7^ indicate a much larger representation of locomotion in human PPC (~660 voxels) in comparison to that of manipulative hand actions (~410 voxels) with the region for skin-displacing actions smaller still (70 voxels). These PPC regions likely represent the sensory stages of the discrimination network, and a larger number of neurons at the sensory stage may improve discriminability, thus boosting the drift rate. However, the correlation between drift rate and accuracy is only partial. This is to be expected, as the proportional-rate diffusion model implies that the latter is equally defined by 2 parameters: drift rate and bound. Earlier studies applying the diffusion model to various tasks demonstrated that model parameters map onto different characteristics of the cognitive processes supporting the task. For example, in recognition memory Ratcliff *et al*.^17^ and McKoon & Ratcliff^18^ report increase in residual time and boundary separation as a function of age. In lexical decision tasks, many aspects of stimuli (e.g. word frequency) could be described by residual time or drift rate^19^. Recently, it was also shown that individual variations in motor style in performing an action affect drift rate in visual action discrimination^13^. In this study, we show, for the first time, that stimulus differences (here the OA classes) influence the bound parameter. These findings are all the more notable that a recent study^20^ failed to find any effect of response modality (pressing keys, pointing or looking), presumed to be closely related to the decision process, on the model parameters of a letter discrimination task.

The dependence of the parameter bound on the observed action class can be better understood if we note that this parameter defines the quantity of information that must be integrated before an observer commits to either of the alternatives. High values describe a conservative slow, but accurate response whereas low values liberal, hasty response^11^. The bound parameter has typically been described in terms of the responding style such as in speed versus accuracy instructions^9,10^, or by testing participants with more or less conservative styles attributed to their age^17,21,22^. It can be speculated that, in the present study, the level of conservatism in decision making reflects less the response style than the ecological validity of observed action classes. Indeed, locomotion, which has the highest bound among the 3 action classes, probably has the most significance for a subject from biological point of view, as knowing the direction and speed of motion of another conspecific can be of vital importance for an organism (to flight or fight). This analysis of kinematic aspects of the actions, however, is required only when the conspecific observed is running, not really when he is walking. Thus the very first thing that needs to be decided is the identity of the OA exemplar, which exactly what we are testing in the present experiments. But any mistake can be costly, which suggests that an accurate decision is critical for an OA class such as locomotion. On the other hand, a manipulative action is rarely perceived in isolation and derives its meaning mostly in the context of the entire chain of manipulations performed by others. It, thus, becomes important to react to a single manipulative action as quickly as possible (to free computational resources for following up actions in the chain), even at the cost of accuracy, yielding the lowest bound among 3 action classes tested.

Initially, single cell recordings suggested that the bound is implemented as a threshold which the firing rate of neurons in the decision region have to reach to trigger a response^23^. This view has been challenged by several recent studies manipulating the speed accuracy trade-off^24^. In particular, Hanks *et al*.^25^ showed that the instruction to respond quickly translates not into a change in the threshold in LIP but results in the addition of a non-evidence dependent, time-varying signal in the direction of the bound, the so called *urgency signal*. A standard decision task, fitted by a stationary model, may use cerebellar signals about available time^26^; However, diffusion models including an urgency signal, as non-stationary models in general, are considered to apply better to tasks with long response times and changing stimuli^27^. It is thus conceivable that a given PPC region devoted to the sensory evidence for a particular action class sends to decision regions not only the fluctuating signals capturing evidence favoring one or the other exemplar of the class, but also a global deterministic signal corresponding to the urgency of response appropriate for that action class. Psychophysical studies show that the optimal height of the bound depends on the belief of the observer about the reliability of the sensory signals, which may vary over the course of the trial^28^, and the time-dependent bias signal may be similar to a biased version of the urgency signal^29^. Bias-like signals have been reported in fMRI studies over parietal and prefrontal regions, particularly in anterior cingulate^30^ and anterior IPS^31^. Such signals may also contribute to the level of the bound. No instructions regarding a speed-accuracy trade-off were given to the subjects in the present study, who were completely naïve as to differences between action classes. It therefore seems that an automatically-generated urgency signal originating in the PPC regions devoted to an action class, is a more likely explanation for our observations. The automaticity of the signal need not to imply a direct connection of PPC regions with the decision stage, as the urgency signal may be computed in frontal regions based on different constraints, and combined with other offset signals before being broadcast to the decision stage.

Drift rate, while differing across action classes, was also responsible for inter-subject variations in thresholds within a class, while bound had no effect on threshold values across subjects. In a between-person comparison, drift rate is typically interpreted as a measure of perceptual sensitivity^9^, reflecting an individual perceptual speed of information processing^32^. The nature of these differences in the processing rate between individual subjects is largely unknown. Several sources of inter individual differences may interact. First, the different drift rates indirectly reflect differences in featural attention to the actions and, as a consequence, different levels of modulation of the OAS neurons representing the sensory stage. This factor may explain the different slopes of the accuracy – drift rate relationships between different classes. Second, there is considerable evidence that cortical thickness underlies inter individual differences in visual perception^33^. This factor would likewise contribute through changing the sensory stage and thus, indirectly, drift rate, although so far no such effect has been reported for action observation. Finally, fluctuation in time of drift rate might provide a third possible explanation for inter-subject differences. Although sensory evidence in a perceptual circuit is generally assumed to be integrated linearly and continuously, recent findings indicate that fluctuations of the drift rate follow the phase of cortical delta oscillations (1–3 Hz) in both lower^34^ and higher^35^ cortical areas. Individual differences in cortical oscillations thus may affect the overall drift rate causing variability in performance within a class. In our previous study^1^, we noted that the duration thresholds for action observation fitted within a single fixation, suggesting that a single cycle of sampling the sensory evidence might suffice to reach a decision. Hence, the contribution of this factor may be minimal.

To conclude, our study has documented clear differences between observed-action classes, both in terms of perceptual performance and of parameters in the diffusion model fitted to the psychophysical data. Further work is needed to understand how the changes in the diffusion model parameters across observed action classes arise. The present results however document a clear stimulus effect on these parameters, particularly the bound parameter, for the first time.

## Materials and Methods

In experiment 1, we tested discrimination for a pair of skin displacing action exemplars (massaging and scratching). In experiment 2, discrimination between two locomotion actions (running and walking) was examined. The methods used were extremely similar to those of our previous study^1^.

### Subjects

Twenty healthy human subjects with normal, or corrected to normal, visual acuity took part in the experiments, ten in experiment 1 (S1–S10) and ten in experiment 2 (S11–S20). This number of subjects per experiment corresponds to the median of sample sizes reported in four APA journals for experimental research^36^. Subjects were naïve as to the purpose of the experiments and gave their informed consent for participation. Experiments followed all national and European guidelines for testing human subjects, and the experimental protocols were approved by the Ethical committee of the Province Parma.

### Setup and Visual stimuli

The same setup was used as in^1^. Subjects were seated 72 cm from a liquid crystal display (Samsung, T27A950, resolution 1920 × 1080 pixels, 50 Hz refresh rate) in an otherwise dark room with their heads supported by a forehead rest and a chin support. The visual stimuli were generated by a personal computer equipped with an open GL graphics card using the Psychophysics Toolbox extensions^37,38^ for Matlab (The Math Works, Inc.). We used a Minolta Luminance Meter LS-100 to calibrate the visual display, with its mean brightness set to 50 cd/m^2^ in all experimental conditions.

In both experiments, video clips (17^o^ × 13^o^, 50 Hz) showing a human actor performing an action, were used as discriminanda. Observers had to discriminate between two action exemplars viewed from the side. This allocentric viewpoint was chosen, because the main interest of the study was observed-action perception. In experiment 1, similarly to Platonov & Orban (2016), the actors were viewed against a background made of a white tissue. In experiment 2, the background was a naturalistic landscape. Video clips lasted 2.6 s with the onset of the action being 10–60 ms from the beginning of the movie and the action continuing till the end of the movie (see temporal profiles in Supplementary Figure 5D). Video margins were blurred with an elliptical mask (14^o^ × 10^o^), leaving the action, the moving body parts and the face of the actor together with the background unchanged but gradually blurring the video into the black background around the edges. In all videos a fixation cross was presented at the same position on the screen.

As before^1^, we created multiple versions of each action exemplar in the two experiments. In experiment 1, 40 versions were generated by combining 2 actors (male, female) × 2 body parts (cheek, chest) × 5 fronto-parallel positions (central plus four positions at 2.5° eccentricity along the diagonals) × 2 sizes (standard, 20% larger). In experiment 2, 48 versions were created by combining 2 actors (male, female) × 2 lateral viewpoints (left, right, with the left viewpoint flipped such that the direction of the observed action would always be from right to left) × 3 fronto-parallel positions (central plus two positions at 2° eccentricity along the vertical) × 2 actor/background interrelationships (actor moving across the screen vs. actor remaining at the point of fixation and background moving) × 2 sizes (standard, 20% larger). In following of Platonov & Orban^1^, we then created for each exemplar version videos with different signal levels (SL) which were set in both experiments to 0%, 6.25%, 12.5%, 25%, 50% and 100%.

To further characterize the videos of the three classes, we analysed local motion following the procedure described in Ferri^7^. The aim of this analysis was to estimate the location of the dynamic changes characterizing the 3 action classes. For each pixel we computed local motion vectors on a frame-by-frame basis^39^. Then, for each video clip, we calculated the number of local motion vectors together with their average magnitude (i.e. speed) by averaging, over frames on the one hand (spatial profile), and over pixels, on the other (temporal profile). Averaging over exemplars after conversion to °/sec, we calculated mean local motion speed temporal and spatial profiles of the three action classes. Supplementary Figure 5 plots mean speed profiles across space (5A-C) and time (5D) for skin displacing, locomotion and manipulative hand action classes (from^1^). Although obviously observed actions cannot be reduced to local motion, the local motion speed is proposed to be proportional to the magnitude of the actions, e.g. the size of the deformations of the body.

### Task

A two-alternative forced-choice (2AFC) action discrimination task was used in both experiments. Subjects viewed a single video clip and indicated, as soon as ready, their choice between two possible exemplars by pressing one of two buttons with the right hand. They had to fixate upon a cross near the center of the screen during each trial. Only this fixation cross was visible during the 2 s inter-trial interval.

A noninvasive monitor-mounted infrared video system (Tobii VersionX2-60) sampled the positions of both eyes at 60 Hz under the control of the Tobii Toolbox extensions of Matlab Version 1.1^40^. Trials contaminated by more than 5% blinks were rejected. In the remaining trials, the standard deviation of the position of the less noisy of the two eye recordings calculated. Fixation performance was similar in all experiments, with the standard deviation averaging 1.13° ± 0.42 horizontally and 0.79° ± 0.30 vertically across the six experiments (Supplementary Table 1).

### Training and test procedures

Before participating in the experiments, all observers were trained in action discrimination. In each experimental group, five subjects were previously trained in manipulative hand action discrimination, whereas the five other subjects were novice in action discrimination. All subjects received equal training (see supplementary information).

Final data were collected in an experimental session including two test blocks of 200 and 240 trials in the first and second experiment respectively. In both experiments, the different videos corresponding to the two action exemplars to be discriminated were presented in random order across trials. To ensure that the subjects correctly remembered the task, the session was preceded by a familiarization block (30 no-noise trials), the results of which were included in the data analysis of experiments as a 100%-signal data point (see *Results*).

### Data analysis

We fitted the data with the proportional-rate diffusion model (following^1,10^), where the bound and drift rate were normalized by the diffusion coefficient reducing to three the number of free parameters: the normalized bound (*A’*), the mean residual time (*t*_*R*_) and the mean drifting rate (*k*).

A significant advantage of a diffusion model over other models is the optimal use of information obtained in the experiment by applying a common metric to assess both accuracy and response time^41,42^. The model predicts that the psychometric function for accuracy *P*_*C*_(*x*) is a logistic function of the percentage of signal *x*:

1 $${P}_{c}(x)=\frac{1}{1+{e}^{-2{A}^{\text{'}}k|x|}}$$

The model prediction for chronometric function of the mean response time *t*_*T*_ *(x)* is

2 $${t}_{T}(x)=\frac{A\text{'}}{kx}\,\tanh (A^{\prime} kx)+{t}_{R}$$

in which percent signal enters the function as both a 1/*x* term and as an argument for the hyperbolic tangent function.

We used the maximum likelihood method to fit the free parameters. In addition to the 75% accuracy thresholds (halfway between chance and perfect performance), we also calculated the halfway response time threshold (midway between the extreme values of the response time curve) and their ratio.

Since the Anderson-Darling goodness-of-fit hypothesis test did not reject the hypothesis that the calculated thresholds were normally distributed, post hoc comparisons of the thresholds were carried out with analysis of variance (ANOVA) and Student’s *t* tests. The results were Bonferroni corrected when necessary.

## Electronic supplementary material

Supplementary Information

Video 1

Video 2
