# Supplementary material for: Not all observed actions are perceived equally
Source: Sci Rep. 2017 Dec 6;7:17084. doi: 10.1038/s41598-017-17369-z (PMC5719070; doi:10.1038/s41598-017-17369-z)
Supplement: Supplementary file 1 — Supplementary Information [file 41598_2017_17369_MOESM1_ESM.pdf]

Supplementary Information:

Not all observed actions are perceived equally

Artem Platonov and Guy A Orban*

Supplementary text:

Training procedure

Before participating in experiments 1-2, all observers received equal training in discriminating between observed massaging and scratching, in experiment 1, and observed running and walking, in the experiment 2. In the beginning, observers had to complete a familiarization block which contained 30 video clips at 100% signal level chosen pseudo-randomly such that a block included 15 (out of 40) versions of the video clips per action exemplar. Observers responded with a button press at the end of a trial and received an auditory feedback with a low-pitched beep-tone indicating a correct response and a high-pitched tone indicating an incorrect response. After subjects learned which button corresponded to which action, they were presented with another familiarization block with feedback to confirm that they could perform the task. If it appeared that the number of errors exceeded 10%, subjects had to perform another familiarization block. The procedure was repeated until the number of errors was 10% or less. If after 5 familiarization blocks, subjects were still unable to perform the task correctly, they did not participate in the experiment.

After completing the familiarization, the subjects were given six 240-trial training blocks (presented in 3 sessions), in which the signal level was manipulated as in the experimental blocks. In addition, each training block was preceded by a familiarization block in which videos without noise were presented. Subjects were given instructions to respond either at the end of the video in the first two training blocks, or as soon as they knew the answer, in the third and subsequent blocks. After finishing training, subjects started with the actual experiment in the next session.

Results

We tested whether the subjects who participated in the previous action discrimination experiment (Platonov & Orban, 2016) performed any differently from totally naïve observers in the two novel OA discrimination tasks. Results from comparing accuracy and half-time thresholds by a multifactor ANOVA indicated no significant differences between the 2 groups (three-way ANOVA: $F_{1,32}=0.33$, $p>0.56$). We, therefore, pooled data from these 2 groups for all further analysis.

One could argue that the skin-displacing actions included a reaching component (~350 ms long), which was much shorter than in manipulative hand actions, while such a component is absent altogether in locomotion. It is unlikely that these differences in temporal structure explain the differences in performance between the action classes. Indeed in skin-displacing videos the information about the action performed after contact with the skin was available already at the

reaching stage. Just like in manipulation, the acting hand was preshaped during the transport phase and this preshaping differed for each of the upcoming actions. Actually, this preshaping started soon after video onset and could thus be used to guide action discrimination. However, to exclude the possibility that our results might be due to an extended period between video and action onset, we remodeled the data from skin displacing actions by subtracting the mean reaching component duration from response times obtained in the experiment. Predictably, this resulted in almost negligible (in the order of 10^{-4}) deviation of the residual time (small increase) and bound (small decrease) parameters from their original values while leaving drift rate completely unaffected. Moreover, this manipulation only increased the difference -though insignificantly- in bound parameter values between skin-displacing and locomotion actions.

Supplementary table 1: *Gaze precision measured as standard deviation (SD) of horizontal and vertical eye positions and percent rejected trials for all experiments.*

Experiment	Subject	Eye Position SD		% Rejected trials
		Horizontal	Vertical	
1	S1	0.97	0.90	80
1	S2	0.60	0.74	18
1	S3	1.25	1.71	63
1	S4	0.92	0.65	24
1	S5	1.01	0.56	19
1	S6	1.11	0.48	13
1	S7	1.29	1.18	16
1	S8	0.86	0.40	9
1	S9	1.07	0.75	6
1	S10	1.60	0.87	27
2	S11	0.92	0.58	16
2	S12	1.13	0.73	26
2	S13	1.14	1.15	28
2	S14	1.84	0.80	31
2	S15	1.87	1.02	36
2	S16	1.06	0.79	26
2	S17	1.05	0.65	8
2	S18	1.17	0.70	4
2	S19	0.99	0.61	3
2	S20	0.77	0.43	16
		1.13±0.32	0.79±0.30	

Supplementary table 2*: Parameter values calculated for proportional-rate diffusion model, fitting the results of the experiment testing manipulative hand actions (rolling/rotation) (from Platonov & Orban, 2016).*

	**Subject**	***A'***	***k***	t_R	**Threshold ratio**	**Threshold (75%)**	**ln(L)**
**Manipulative hand actions**	S1	1.53	28.7	0.30	3.50	12.5	23.6
	S2	1.45	27.1	0.33	3.49	14.0	15.8
	S3	1.22	37.8	1.14	3.50	11.9	12.1
	S4	1.26	31.6	0.76	3.49	13.8	15.4
	S5	0.78	49.1	1.06	3.49	14.4	14.8
	S6	1.38	16.9	0.13	3.49	23.7	5.71
	S7	0.93	36.0	0.76	3.48	16.5	9.96
	S8	1.51	19.5	0.04	3.49	18.7	5.89
	S9	1.25	21.1	0.43	3.49	20.9	24.8
	Mean (SD)	1.11 (0.30)	29.8 (10.2)	0.55 (0.40)	3.49 (0.01)	16.3 (4.04)	

Supplementary table 3*: Parameter values calculated for proportional-rate diffusion model, fitting the results of experiments 1 and 2 (A' = normalized bound; k = drift rate;* t_R *= mean residual time in s), threshold ratio, estimated 75% accuracy threshold and quality of fit (L =likelihood). The* $\chi 2$ *is based on a likelihood ratio test comparing reduced to original fuller model. *p<0.05*

	Subject	*A'*	*k*	t_R	Threshold ratio	Threshold (75%)	ln(L)	$\chi^2(2)$
Experiment 1	S1	1.68	16.6	0.16	3.48	19.6	21.6	0.41
	S2	1.25	27.1	1.05	3.48	16.2	27.8	2.24
	S3	1.51	18.9	0.38	3.48	19.3	22.5	1.14
	S4	1.59	15.6	0.24	3.49	22.1	20.4	3.19
	S5	2.04	9.9	0.93	3.48	27.1	12.8	0.48
	S6	1.77	22.9	0.30	3.48	13.6	18.5	20.0*
	S7	1.43	13.1	0.63	3.48	29.5	42.6	0.12
	S8	1.07	35.3	1.17	3.49	14.6	25.9	0.87
	S9	1.57	15.0	0.32	3.49	23.3	32.5	2.06
	S10	1.95	12.3	0.39	3.49	22.8	39.4	0.99
	Mean (SD)	1.59 (0.30)	18.7 (7.80)	0.56 (0.37)	3.48 (0.01)	20.8 (5.19)		
Experiment 2	S11	1.99	21.0	0.76	3.49	13.2	9.54	50.6*
	S12	2.19	12.7	0.47	3.49	19.8	15.7	9.90*
	S13	1.72	33.9	1.91	3.50	9.4	21.0	70.7*
	S14	2.02	19.7	0.48	3.48	13.8	10.5	71.7*
	S15	1.96	21.0	0.70	3.49	13.3	15.4	43.1*
	S16	2.13	24.0	1.03	3.50	10.7	19.0	33.9*
	S17	1.90	18.0	0.42	3.49	16.0	21.4	96.7*
	S18	2.13	21.9	0.43	3.47	11.8	11.4	84.2*
	S19	1.93	19.9	0.77	3.49	14.3	29.4	82.2*
	S20	2.06	26.4	1.25	3.48	10.1	10.0	43.6*
	Mean (SD)	2.00 (0.14)	21.9 (5.57)	0.82 (0.47)	3.49 (0.01)	13.2 (3.08)		

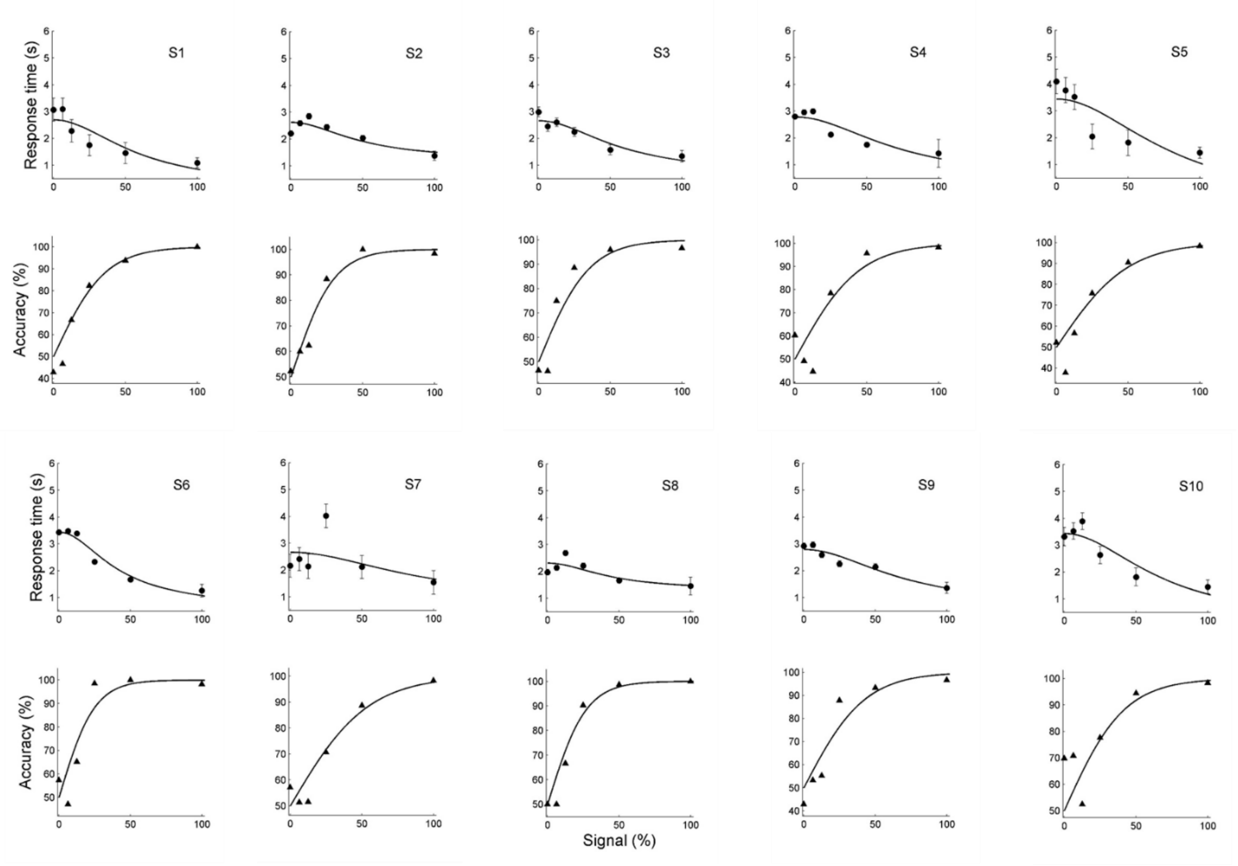

Supplementary Figure 1: *Experiment 1: Response time (circles, upper rows) and accuracy (triangles, lower rows) plotted as a function of signal strength for 2AFC discrimination of skin-displacing actions (scratching/massaging) by subjects S1-S10. The proportional-rate diffusion model provided a close fit (solid lines) to the data in all subjects. Error bars indicate ±1 SEM.*

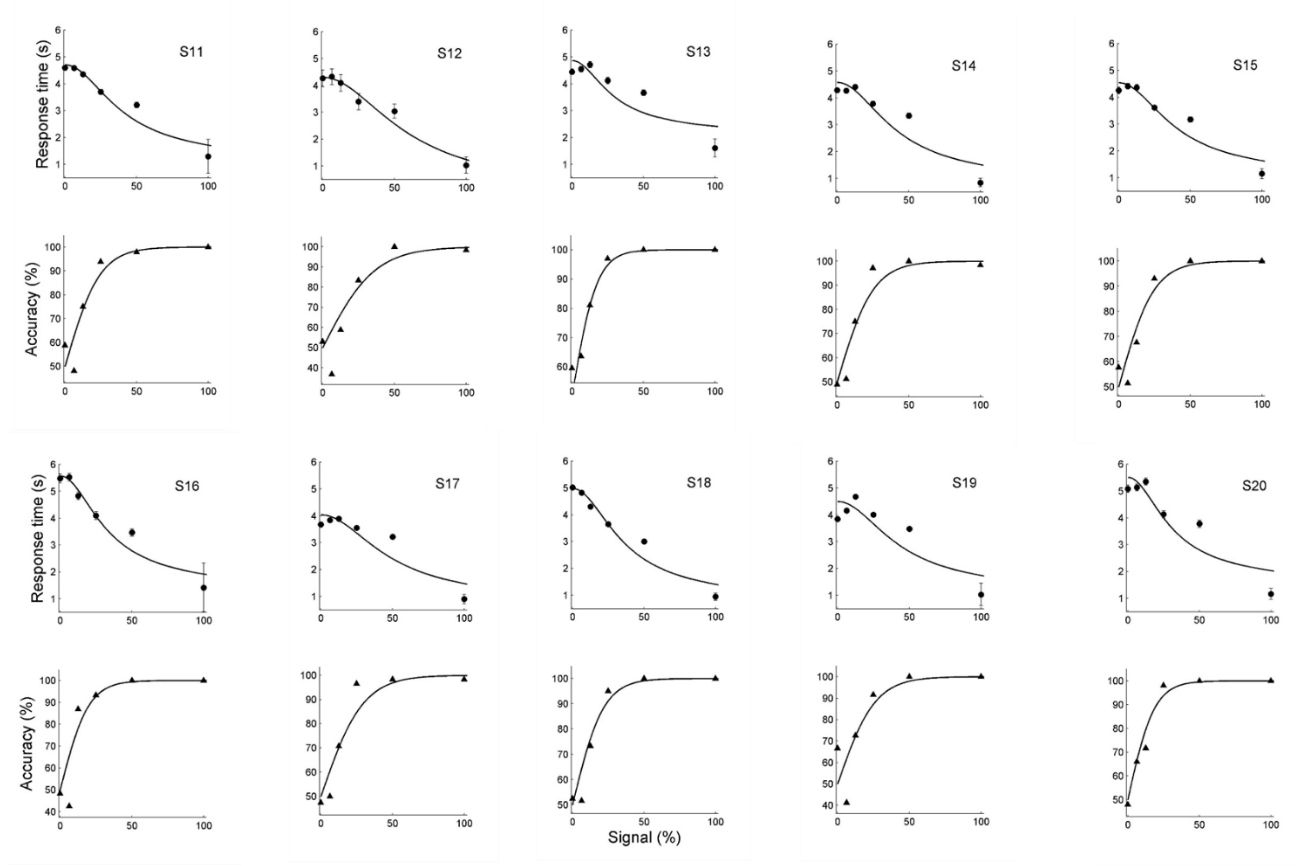

Supplementary Figure 2: *Experiment 2: Response time (circles, upper rows) and accuracy (triangles, lower rows) plotted as a function of signal strength for 2AFC discrimination of locomotion actions (running/walking) by subjects S11-S20. The proportional-rate diffusion model provided a close fit (solid lines) to the data in all subjects. Error bars indicate ±1 SEM.*

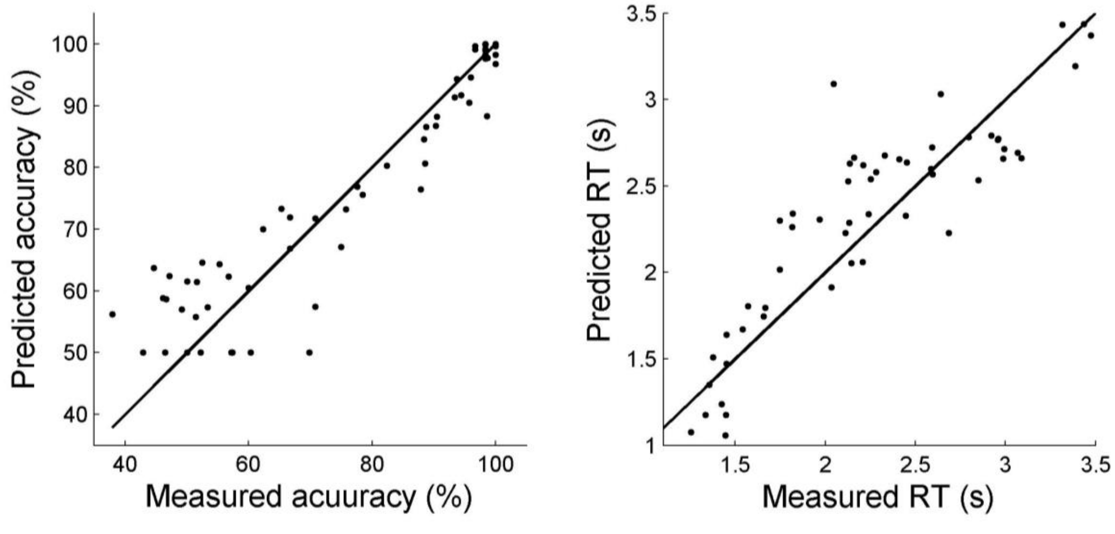

Supplementary Figure 3: *Scatterplot of predicted versus measured accuracy (A) and response time (B), obtained in the experiment 1. Plot shows that both datasets were well-described by proportion-rate diffusion model (Main text). Dots identify the results from individual subjects.*

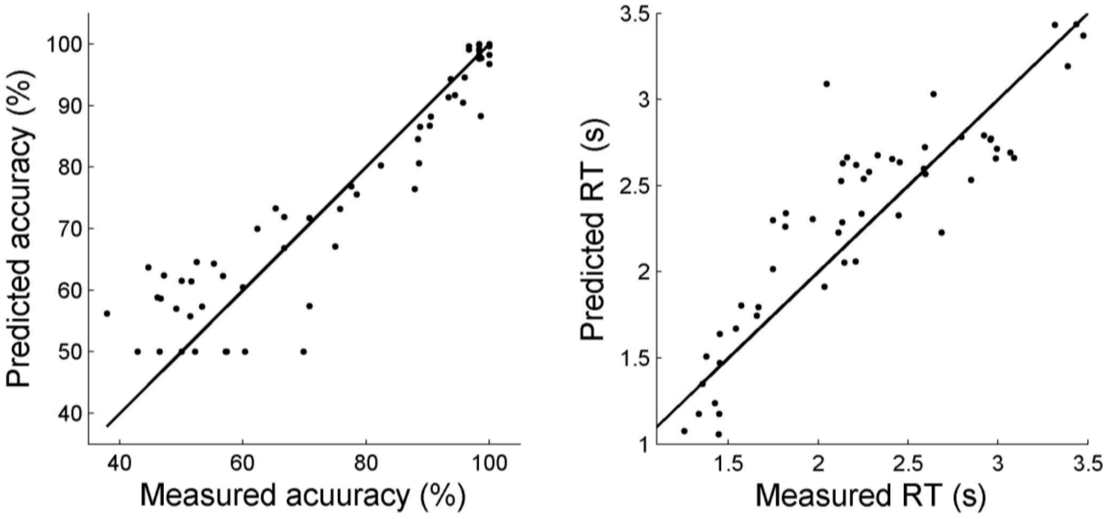

Supplementary Figure 4: *Scatterplot of predicted versus measured accuracy (A) and response time (B), obtained in the experiment 2. Dots identify the results from individual subjects.*

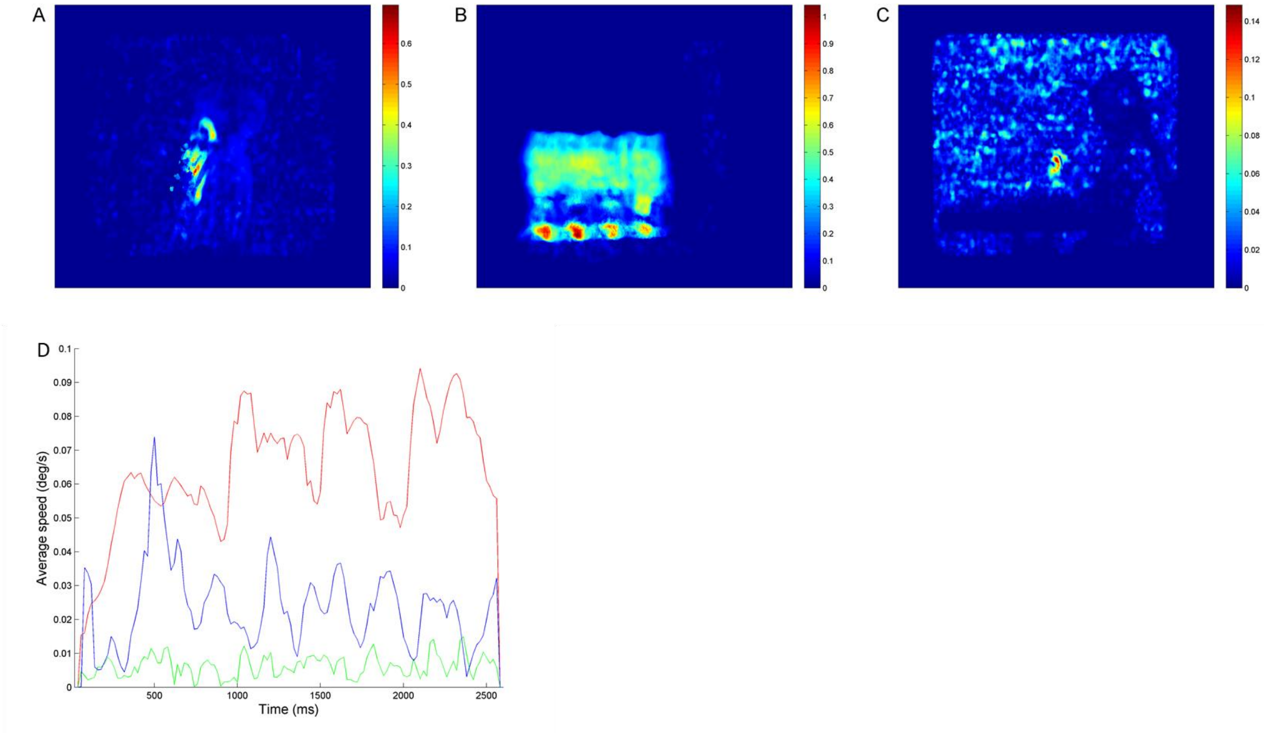

Supplementary Figure 5: *A-C: Local motion speed (°/s) averaged over time for skin displacing (A), locomotion (B) and manipulative hand (C) actions The number of local motion vectors in locomotion actions exceeded that in skin displacing and manipulative hand actions by factors of about 1.5 and 3, respectively. The amplitude in locomotion actions was about 3 and 10 times greater than in in skin-displacing and manipulative hand actions, respectively. The amount of local motion in skin-displacing actions was 2 times greater in terms of the number of local motion vectors and more than 3 times greater in terms of magnitude (i.e. speed) if compared against manipulative hand actions. E: Local motion speed (°/s) averaged over space for skin displacing (blue), locomotion (red) and manipulative hand (green) actions.*
